# Determinants of enrolment for community-based health insurance in Somali region of Ethiopia

**DOI:** 10.4102/jphia.v16i1.726

**Published:** 2025-02-18

**Authors:** Abdifatah Elmi, Olusola Oladeji, Ahmed Tahir

**Affiliations:** 1 Health Section, UNICEF Somali Field Office, Jigjiga, Ethiopia; 2 School of Public Health, Jigjiga University, Ethiopia

**Keywords:** health insurance, determinants, enrolment, agro-pastoral, Somali region, Ethiopia

## Abstract

**Background:**

Community-based health insurance (CBHI) is a type of health insurance programme that provides financial protection against the cost of illnesses and improves access to healthcare services for communities in the informal sector.

**Aim:**

The aim was to assess the determinants of enrolment for CBHI in Aw-barre district – an agro-pastoral setting in the Somali region, Ethiopia.

**Setting:**

Ethiopia launched the CBHI scheme in 2011 as part of the revised healthcare financing strategy to realise universal health coverage. It was then scaled up in the rural part of the country in 2013, except the pastoralist regions including the Somali region which started as late as 2020.

**Methods:**

A community-based, unmatched case-control study was conducted using a concurrent nested approach between March 2021 and April 2021, among 214 participants (54 enrolled and 160 non-enrolled). Quantitative data were analysed using SPSS version 20, and thematic analysis was performed for the qualitative data.

**Results:**

Awareness of the CBHI scheme adjusted odds ratio (AOR) = 9.67 (1.26, 74.53), household income AOR = 3.56 (1.03, 12.30) and being a member of community-based solidarity groups AOR = 2.48 (1.17, 5.26) were the determinants for CBHI enrolment and were reaffirmed by the qualitative findings.

**Conclusion:**

Increasing community awareness of the scheme via various platforms is essential. Leveraging community-based solidarity associations, and social protection platforms would help increase enrolment.

**Contribution:**

Given the distinct sociodemographic, economic and geographic peculiarities of agro-pastoralists, the CBHI parameters and implementation strategies must be tailored to the setting before scaling it up.

## Introduction

Universal health coverage means that all people have access to the health services they need when and where they need them without financial hardship.^1^ Progress towards this aspiration seems poor, particularly for countries whose fiscal capacity is low and whose social health insurance for the employed sector is absent or very small, thus limiting the mobilisation of additional resources from payroll contributions.^2^

Financing healthcare in most developing countries relies greatly on out-of-pocket payments, which contributes to an unacceptably high burden of preventable diseases and deaths.^3^ Developing countries often have a higher proportion of households experiencing catastrophic health expenditure, which includes spending over 40% of consumption expenditure on health.^4^

Ethiopia envisioned to ensuring universal health coverage through primary health care by 2035.^5^ The per capita spending is 36.3% far below the globally recommended $86 per capita, which is estimated to make essential healthcare services available in low-income countries. The government spends 6.3% of its budget on the health sector, which is below the Abuja target of 15%, and the out-of-pocket expenditure is still high, at 30% of total health expenditure.^6^

Community-based health insurance (CBHI) is a type of health insurance programme that provides financial protection against the cost of illness and improves access to healthcare services for communities in the informal sector.^1^

The available evidence demonstrates that health insurance can be an alternative to user fees as a health financing mechanism and can improve financial protection and service utilisation patterns.^7,8^ However, there is weaker evidence that health insurance can foster social inclusion of specific vulnerable groups in low- and middle-income countries (LMICs).^9^

In Ethiopia, the CBHI scheme was launched and piloted in 2011 to contribute to the reduction of outpocket payments and realisation of universal health coverage.^10^ Approximately 1006 districts out of the estimated 1246 districts in the country have implemented the programme corresponding to 81% geographic coverage nationwide against the 2020 target of 80% CBHI coverage in all districts. Most of these districts are concentrated in four agrarian regions and the nation’s capital city.^11^

The Somali region among Ethiopia’s four Developing Regional States (DRS) is predominantly inhabited by pastoralists (85%) who face developmental inequities and lagging in key health outcomes compared to the national average.^12^ The insurance scheme was first launched in 2020 in Aw-barre district, an agro-pastoral setting, along with three other districts with more than 31% of the eligible households registered – a very low enrolment rate – unlike the national target coverage of 80% set for districts.^13^ Thus, this study aimed to identify the determinant factors for CBHI scheme enrolment in the district and to inform strategies to improve the enrolment rate of the scheme in the region.

## Research methods and design

### Study setting

The Aw-barre district is 72 km North of Jigjiga, the capital of the Somali region of Ethiopia, and is one of the first Districts to start CBHI implementation in the Somali region of Ethiopia. Administratively, the district comprises of 44 villages (kebeles) arranged in six CBHI clusters and its population is projected to be 382 569 based on the 2007 national census conducted by the Central Statistical Agency of Ethiopia. The number of households enrolled in the CBHI scheme was 10 100, which roughly translates to a 16% registration rate (district estimation). This study was conducted between 24 March 2021 and 24 April 2021.

### Study design and population

This community-based, unmatched case-control study (enrolled and non-enrolled members) used a concurrent nested approach of quantitative and qualitative methods.

### Source and study populations

The source population consisted of all households residing in the Aw-barre district, at least in the last 6 months before the study, and the study population included the selected households enrolled (cases) in the scheme and those not enrolled in the scheme (controls). Household heads who did not consent to be interviewed were excluded.

### Sample size estimation

The sample size was computed using Epi Info™ 7 software StatCal with the following assumptions: 80% statistical power with a level of significance at 5%, odds ratio of 4.16, percentage of cases exposed 77 (95.1%), percentage of controls exposed to 472 (85.7%)^12^ and a case-control ratio of 1:3. This has resulted in a final sample size of 214 (54 enrolled and 160 non-enrolled).

For the qualitative part, considering the homogeneity of the population and convenience for moderation, four focus group discussions (FGDs), each consisting of 6–12 participants, and three key informant interviews (KIIs) were conducted based on information saturation.

### Sampling methods and procedure

**FIGURE 1:**
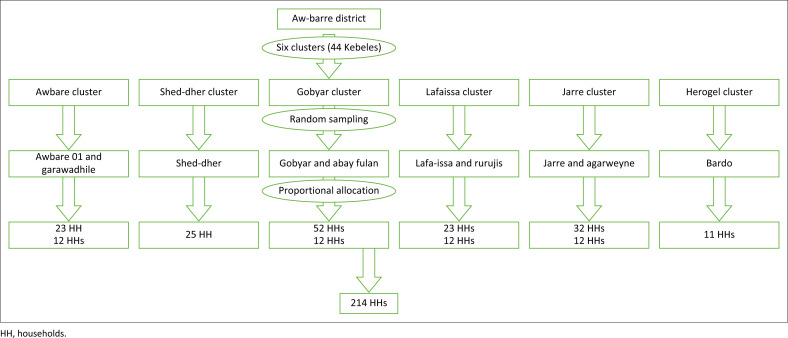
Schematic presentation of the sampling procedure.

The quantitative part of the study was conducted using a multistage cluster-sampling technique. Ten kebeles (villages) were selected by simple random sampling in the first stage. In the second stage, a probability proportional to the size of the households is used to allocate households across selected village or kebele. The cases and controls were chosen using the lottery method (Figure 1).

In the qualitative part, purposive sampling was employed to collect information from stakeholders involved in the execution of the scheme. Four FGDs were performed (two respectively for enrolled and non-enrolled households). Key informants from the district health office representative, the district CBHI coordinator and the Regional Health Bureau focal point for CBHI were interviewed. All interviews were conducted face-to-face in convenient places using an interview guide designed to extract the participants’ viewpoints using open-ended questions with probes to provoke additional discussions.

### Data collection tools and techniques

Structured questionnaire was adopted from the national evaluation of CBHI^15^ and other literature^14,16,17^ and was administered by trained enumerators to the heads of selected households. Two semi-structured interview guides were used for the FGDs and KIIs both were administered by the interviewers.

### Data quality assurance

Training was provided to the data collectors and supervisors for 2 days to ensure data quality. The questionnaires were reviewed daily for completeness and consistency by the supervisors and the principal investigator. All necessary feedback was provided to the data collectors the following morning before resuming data collection.

### Data analysis

Data were entered and analysed using Statistical Package for Social Sciences (SPSS) version 20. Multivariate logistic regression analyses were used to control for potential confounding variables,^18^ and the final determinant variables for enrolment in the CBHI scheme were reported using adjusted odds ratio (AOR) at 95% CI and *p*-value < 0.05.

As to the qualitative data, field notes were taken, and the entire discussion was audio-recorded in the local language, transcribed verbatim and translated into English. Emerging themes were summarised thematically, and quotes from participants were presented contextually. The quantitative and qualitative results were integrated in the discussion/interpretation phase using triangulation technique.

### Ethical considerations

An ethical clearance letter with reference number IHRRC039/2021 was obtained from the research Ethics committee of the Institute of Health Sciences, Jigjiga University, Ethiopia. Oral consent was secured before administering the questionnaire to the participants.

## Results

### Socio-demographic profile of respondents

Table 1 shows the 214 households that participated in the study (54 cases and 160 controls) with a 100% response rate. Among the participants enrolled in the scheme, 14 (24.1%) were from rural households and 41 (75.9%) were from urban households. Most of the enrolled household heads had no formal education 33 (61.1%) and more than six household members 31 (57.4%) (Table 1).

**TABLE 1:** Socio-economic and demographic characteristics of respondents, Awbarre district, Somali region, Ethiopia 2021 (*N* = 214).

Variables	CBHI enrolment			
	No (*n* = 162)		Yes (*n* = 54)	
	*n*	%	*n*	%
**Age of household head (years)**				
18–30	36	22.2	14	25.9
31–40	65	40.1	24	44.4
41–50	38	23.5	9	16.7
> 50	23	14.2	7	13.0
**Sex of household head**				
Female	81	50.0	29	53.7
Male	81	50.0	25	46.3
**Marital status of household head**				
Divorced/widowed	11	6.9	1	1.8
Married	136	85.5	52	91.2
Single	12	7.5	4	7.0
**Educational status**				
No formal education	120	74.1	33	61.1
Primary	37	22.8	14	25.9
Secondary/post-secondary	5	3.1	7	13
**Occupation**				
Agro-pastoralist	91	56.2	25	46.3
Daily laborer	42	25.9	15	27.8
Merchant	26	16.0	12	22.2
Other	3	1.9	2	3.7
**Place of residence**				
Rural	53	32.7	13	24.1
Urban	109	67.3	41	75.9
**Elderly person above 65 years in household**				
No	115	71.0	40	74.1
Yes	47	29.0	14	25.9
**Household Size**				
< 3	8	4.9	2	3.7
3–4	23	14.2	10	18.5
4–6	44	27.2	11	20.4
> 6	87	53.7	31	57.4
**Annual household income (ETB)**				
< 8000	87	53.7	20	37.0
8001–16 000	54	33.3	14	25.9
16 001–28 000	12	7.4	8	14.8
> 28 000	9	5.6	12	22.2

CBHI, community-based health insurance; ETB, Ethriopian birr.

The result of bivariate analysis in Table 2 shows that households with information about CBHI were more likely to enrol in the CBHI scheme compared to those with no information crude odds ratio (COR) = 12.7 (1.67, 95.7). Being a member of a local solidarity group like a local money-saving association was more likely to go for CBHI enrolment COR = 2.23 (1.15, 4.31). Also, households who considered the premium (preset payment of 250 ETB [Ethriopian birr]) as affordable were more likely to enrol in the CBHI, COR = 2.7 (1.1, 6.9) as shown in Table 2.

**TABLE 2:** Bivariate analysis of household characteristics of respondents and CBHI enrolment, Awbarre district, Somali region, Ethiopia April 2021 (*N* = 214).

Variables	CBHI enrolment				COR	95% CI	*p*
	No (Control)		Yes (Case)				
	*n*	%	*n*	%			
**Heard of CBHI?**							
No	31	19.4	1	1.9	1.00	-	-
Yes	129	80.6	53	98.1	12.70	1.67, 95.7	0.013
**Perceived affordability of the premium (250 ETB) per year**							
No	41	25.6	6	11.1	1.00	-	-
Yes	119	74.4	48	88.9	2.70	1.1, 6.9	0.031
**Enrolled in a solidarity group**							
No	124	77.5	33	61.1	1.00	-	-
Yes	36	22.5	21	38.9	2.23	1.15, 4.31	0.018
**Trust on the CBHI scheme?**							
No	20	12.5	4	7.4	1.00	-	-
Yes	140	87.5	50	92.6	1.76	0.57, 5.40	0.323
**Chronic illness in the Household?**							
Does not exist	125	78.1	37	68.5	1.00	-	-
Exists	35	21.9	17	31.5	1.61	0.81, 3.19	0.173
**Time to reach the nearest health facility?**							
< 30 minutes	128	80.0	51	94.4	5.53	0.71, 43.19	0.103
> 60 minutes	18	11.2	2	3.7	1.47	0.12, 17.91	0.761
30–60 minutes	14	8.8	1	1.9	1.00	-	

CBHI, community-based health insurance; ETB, Ethriopian birr; COR, crude odds ratio.

### Determinants of community-based health insurance enrolment in Aw-Barre district

Table 3 shows the results of the multivariate analysis. The variables considered in the multivariate logistic regression model were all those with a *p*-value < 0.25 at the bivariate analysis level.

**TABLE 3:** Determinants of CBHI enrolment using multivariable analysis in Aw-barre district, Somali Region, Ethiopia, April 2021 (*N* = 214).

Variables	CBHI enrolment				AOR	95% CI	*p*
	No (Control)		Yes (Case)				
	*n*	%	*n*	%			
**Heard of CBHI?**							
No	31	19.4	1	1.9	1.00	-	-
Yes	129	80.6	53	98.1	9.67	1.26, 74.53	0.029
**Perceived affordability of the premium (250 ETB) per year**							
No	41	25.6	6	11.1	1.00	-	-
Yes	119	74.4	48	88.9	1.98	0.75, 5.22	0.166
**Enrolled in a solidarity group**							
No	124	77.5	33	61.1	1.00	-	-
Yes	36	22.5	21	38.9	2.48	1.17, 5.26	0.017
**Household Income in ETB**							
< 8000	87	54.4	20	37.0	1.00	-	-
8001—16 000	54	33.8	14	25.9	0.73	0.31, 1.75	0.484
16 001 –28 000	12	7.5	8	14.8	2.73	0.77, 9.57	0.118
> 28 000	7	4.4	12	22.2	3.56	1.03, 12.30	0.044

CBHI, Community-based health insurance; ETB, Ethriopian birr; AOR, adjuster odds ratio.

At the multivariate level, households with high income, being members of a solidarity group and having awareness about the CBHI scheme were found to be determinants of CBHI enrolment.

Households with better incomes were almost four times more likely to enrol in CBHI than low-income households (AOR = 3.56 [1.03, 12.30], *p* = 0.044). Similarly, households with no information about CBHI were nine times more likely to enrol than those with no information (AOR = 9.67 [1.26,74.53], *p* = 0.029). Moreover, households that were members of a solidarity group like saving associations or other community-based organisations were almost 2.5 times more likely to enrol than non-members (AOR = 2.48 [1.17, 5.26], *p* = 0.017).

### Findings from the qualitative part

Four FGDs were conducted – two from each CBHI-enrolled and non-enrolled households, respectively, in rural and urban kebeles.

Three main themes emerged from the interviews and FGDs (1. Reasons for enrolment & non-enrolment, 2. Indigents targeting, 3. Success & challenges), and the first theme has two sub-themes: *awareness & affordability*. For this article, the first and the third themes were considered for discussion.

The common reason for joining the CBHI was the need to obtain accessible healthcare services free of charge for family members:

‘I registered to get medicines and free health care services for my family.’ (CBHI member in the Lafa-issa kebele of Awbarre woreda, 39 years old women with children)

For non-CBHI members, the most common reason for not being enrolled was not having heard of CBHI, followed by unaffordable payments:

‘It is through this FGD session, that I first hear of community-based health Insurance scheme.’ (Non-CBHI member in the Lafa-issa kebele of Awbarre woreda, Business women, 43 years old)

Regarding indigent targeting, FGD members from different villages have varied views. While some FGD members claimed that the poor were identified reasonably in their villages and their payments were covered, other FGD members from the other villages claimed that indigent identification and targeting did not occur:

‘Membership registration in this kebele was based only on the ability to contribute 250 Birr, no single indigent was registered.’ (CBHI member in Abayfulan Kebele, Awbarre District, 48 years old, Male)

‘The selection process for the poor households was transparent and fair in my view. I think the process is based on the economic severity of households. I can say needy households were not left out.’ (CBHI member in Lafaissa, Aw-Barre District, Women, 45 years old)

Key informants at the regional and district levels described the commendable progress of the scheme within a short period and the challenges that came along with it:

‘Some of the major achievements include helping the community understand the benefits of insurance, managing many poor households and people with chronic illnesses to get enrolled into the scheme and receive free service including laboratory services. But some challenges such as community misunderstandings require further work.’ (Awbarre woreda, CBHI coordinator, Male)

## Discussion

This study identified determinants of enrolment for CBHI in an agropastoral district of the Somali region, Ethiopia. This includes awareness about CBHI, household income, perceived affordability of the premium and being a member of a solidarity group.

This study found that having information about CBHI was a key determinant of enrolment in the scheme. Households with information about CBHI were more likely to get enrolled in the scheme. The poor enrolment might be related to inadequate sensitisation at the community level given the early implementation stage, which is also affirmed by some FGD participants in the study.

Similar findings were reported in Ethiopia,^14,15,16,19,20^ Nigeria,^21^ Uganda^22^ and Cameroon,^23^ which identified inadequate knowledge and understanding of insurance as obstacles to enrolment. Inadequate client education and limited community engagement threaten the scheme’s sustainability.^24^ Similarly, another study in Ethiopia found that knowledge of CBHI not only determines the enrolment but is also associated with a dropout from the scheme.^25^

This study additionally found that membership in a solidarity group is a significant determinant in household enrolment increasing the likelihood of participation in the scheme. Community solidarity is a type of social capital at the community level that influences households’ decisions in favour of health insurance, increasing the demand and sustainability of CBHI and providing a platform for CBHI scheme sensitisation.^23,26,27,28,29^ This is consistent with studies indicating that trust and solidarity motivate groups to pool their resources for common use to share risks.^16,19,23^

Another determinant identified to affect CBHI enrolment is household income. Households with higher yearly incomes tended to have enrolled in the scheme; this is consistent with other studies in various settings.^16,20,22,29,30,31^ Lower levels of income were found to be among the main barriers to enrolment according to systematic reviews in LMICs.^32^ Moreover, household income impacts on affordability positively correlating with the willingness to pay the premium.^10,30,31,32,33^ This result is comparable to the results of Ethiopia’s CBHI evaluation, which revealed that 39% of families cited the cost of registration fees and premiums as a barrier.^15^ Flat-rate premiums were found to harm the poor’s decision to enrol according to research findings in Ghana, Nigeria, Mali and Senegal.^32,34,35,36^

Contrarily, a prior study in Ethiopia indicated that most food-insecure households were substantially more likely to enrol and that the socioeconomic level of the household does not limit the adoption of CBHI.^37^ This study was done in a scenario where low-income families participated in a safety-net programme that targets families with persistent food insecurity. Households that are recipients of both CBHI and productive safety net programmes saw an increase in livestock by 5% and a decrease in debt by 27% providing vulnerable households protection against risks.^38^

The qualitative findings of this study indicated that accessibility of enrolled households to health facilities has increased, mainly attributed to non-payment at the point of care for family members. Low-income rural and urban households’ access to services was enhanced by protecting members financially against the out-of-pocket expenses of medical care. This is supported by data from one of the study area’s health centres (Awbarre health center), which showed an increment of patient visits jumping from 4560 in 2020/2021 to 11 200 in 2021/2022 following the commencement of CBHI service in October 2021.^13^ On the other hand, non-enrolled households indicated information unavailability and unaffordability of the premium as common reasons for their non-enrolment. In line with this, the affordability of premiums and the registration cost were also indicated by 39% of non-members as challenges, according to the CBHI evaluation in Ethiopia.^15^

Key informants in this study have acknowledged the key accomplishments made so far in terms of implementation of the scheme as well as the challenges encountered. In the agro-pastoralist context, significant segments of the population are on the move, settlements are scattered and health facilities are limited and not evenly distributed. Despite the differing environments and lifestyles, agro-pastoralist districts have implemented the CBHI programme using the same design criteria as sedentary farming areas.^39^ On the same note, health financing structures are not in place at all levels of the health system in pastoralist regions, and the implementation and institutionalisation of first-generation reforms like health facility governance boards, as well as revenue retention and utilisation, are suboptimal.^39^ Furthermore, existing obstacles related to physical access, distance to health facilities and cultural views about the ineffectiveness of particular medical treatments might discourage some from joining CBHI; on top of the inadequate readiness of existing health facilities and poor quality of service provision because of personnel, medications and laboratory services.^40,41,42^

The result of this study was dependent on the feedback given by the respondents, which might have been subjected to respondent bias and recall bias. The study’s strength is the use of a mixed methods approach, which helped in the triangulation of the information and provided better clarity and understanding of the quantitative data.

## Conclusion

Knowledge of the insurance scheme, household income and membership in a local solidarity group determines household enrolment. Increasing community awareness using appropriate community platforms is critical. Leveraging on the existing community-based solidarity associations and other social protection platforms would help increase enrolment for the scheme.

### Contribution

Because pastoralists and agro-pastoralists have distinct sociodemographic, economic, and geographic traits, the CBHI parameters and implementation strategies should be appropriate for the context before being scaled up.
